# The influence of physical exercise on loneliness of college students: mediating and moderating roles

**DOI:** 10.3389/fpsyg.2025.1631623

**Published:** 2025-08-15

**Authors:** Yadong Liang, Lijun Wang, Xingbin Du

**Affiliations:** ^1^School of General Education, Shandong Huayu University of Technology, Dezhou, China; ^2^Institute of Physical Education and Health, Yulin Normal University, Yulin, China

**Keywords:** college students, a sense of loneliness, physical exercise, emotional regulation self-efficacy, mental resilience

## Abstract

**Objective:**

To explore the relationship between physical exercise and loneliness of college students and its mechanism, and to analyze emotional self-efficacy as a mediating variable and psychological elasticity as a moderating variable.

**Methods:**

In this study, the Physical Exercise Scale, the UCLA Loneliness Scale, the Emotion Regulation Self-Efficacy Scale (RES) and the psychological elasticity Scale were selected and used in a cross-sectional design to conduct a comprehensive survey of 850 college students from five different schools, for variables such as gender, grade, major, and place of origin. In the subsequent stage of data processing and analysis, SPSS 27.0 was used to descriptive statistics and correlation analysis to carry out a series of processing and analysis work on the collected relevant data. On this basis, in order to explore the mediating role of emotion self-efficacy in the relationship between physical exercise and loneliness more deeply, the researchers further constructed a structural equation model using AMOS28.0 software. The mediation effect ERSE and its 95% confidence interval (CI) were tested using the bootstrap sampling method (5,000 times), which is mainly used to detail the path between variables.

**Results:**

There was a significant correlation between physical exercise, loneliness, emotional regulation self-efficacy, and psychological elasticity. The results of the direct effect analysis showed the direct predictive effect of physical exercise on the loneliness of college students was significant (*β* = −0.600, *p* < 0.001). The of the mediation effect analysis showed that the standardized coefficient of the path “physical exercise → emotional regulation self-efficacy” was 0.580 (*p* < 0.001), and the standardized coefficient of the path “emotional regulation self-efficacy → loneliness” was −0.474 (*p* < 0.001); psychological elasticity played a partial mediating role between physical exercise and loneliness in college students (*β* = −0.156, *p* < 001).

**Conclusion:**

Physical exercise can not only positively affect loneliness of college students, but also mediate and regulate self-efficacy and mental resilience through emotional regulation, respectively.

## 1 Introduction

Loneliness refers to the subjective psychological experience generated when an individual feels an unpleasant interpersonal relationship and has a large gap with his actual communication level, which is usually accompanied by negative emotions such as isolation, helplessness and emptiness (Ma and An, 2019). Every individual has experienced the feeling of loneliness, and loneliness has gradually become a common phenomenon in society. College students are in the special growth stage and psychological development stage, the experience of loneliness is particularly profound. Among them, the first and third years of college are the high incidence of loneliness (Cheng and Yang, n.d.). Recent studies have also shown that loneliness, as a modern “epidemic,” first peaks around adulthood (Lee et al., 2018). If you are in negative emotions like loneliness for a long time, it is easy to cause a series of emotional disorders, which is easy to cause great harm to physical health (Yang et al., 2022). Moreover, loneliness will make individuals feel distant from others or society, which will lead to personality disorder over time (Cacioppo et al., 2015). Therefore, this paper studies the mechanism of loneliness in college students, improves the level of mental health of college students, and provides a theoretical basis for preventing and intervening loneliness in college students. College students are in a critical period of psychosocial development, facing multiple pressures from academics, social interactions, and future planning. Loneliness, a prevalent and significantly harmful psychological experience, has become an important risk factor affecting their mental health and life quality (cite relevant studies, such as post-COVID-19 research or-term trend data). In the quest for effective psychological intervention strategies, exercise has been widely regarded in the fields of sports psychology and public health as a potentially key avenue for enhancing health due to its accessibility and minimal side effects. However, the specific mechanisms and effects of how exercise influences loneliness, a core subjective experience reflecting the quality of an individual’s connections, remain to be thoroughly explored, which is the central question this study aims to address.

As the saying goes, “Exercise is a good medicine for all diseases.”Physical exercise can regulate the individual’s psychological emotions, often participate in physical exercise can make people maintain a healthy mental state. This study was based on social cognitive theory and the biopsychosocial model, and constructed an integrated framework of the effect of physical exercise on loneliness. The cognitive theory emphasized the core role of self-efficacy in the relationship between behavior and emotional outcomes, and held that individuals’ beliefs about their capabilities directly affect their motivation, behavioral, and emotional states. At present, physical exercise and loneliness of college students are closely related, and the role of physical exercise in alleviating loneliness has been confirmed by many scholars. For example, scholar Zhong Wei’s research results such as show that physical exercise can improve the loneliness symptoms of college students by improving social self-efficacy (Zhong et al., 2019). Han Chaoyi et al. found that individuals who often participate in physical exercise have a lower sense of loneliness, and physical exercise can regulate the psychological state of individuals (Han et al., 2019). Wang Shiying conducted exercise intervention on individuals and found that individuals’ sense of loneliness was significantly improved after a period of intervention (Wang, 2019). In addition, a research survey shows that college students who do not participate in physical exercise will have a serious problem of loneliness, indicating that physical exercise is significantly correlated with loneliness, which indicates that physical exercise can be used as an effective indicator to measure loneliness (He and Xu, 2002). Therefore, hypothesis H1 is proposed in this paper: physical exercise can negatively predict loneliness of college students. This hypothesis stems from a fundamental consensus in sports psychology: that physical activity itself can promote the release of endorphins, improve mood tone, and provide opportunities for interaction, thereby potentially directly mitigating an individual’s experience of loneliness.

Although physical exercise may directly improve negative emotions such as loneliness, are there other mediating variables affecting the relationship between the two? Emotional regulation self-efficacy refers to the strength of an individual’s belief in their ability to identify, understand, and regulate positive and negative emotions. It encompasses three dimensions: expressing positive emotions (POS), regulating distress and pain (DES), and regulating anger (ANG). High RES individuals tend to actively use adaptive emotional strategies (such as cognitive reappraisal) and exhibit greater psychological elasticity when experiencing emotional distress. Emotional regulation self-efficacy refers to the adjustment and improvement of an individual’s psychological and emotional state (Qi and Yu, 2018). Previous studies have found that loneliness is a negative emotion, and emotional self-efficacy plays a mediating role in the relationship between physical exercise and mental health of college students (Qi and Yu, 2018). Emotional Regulation Self-Efficacy directly stemmed from Bandura’s core concept of self-efficacy. Self-efficacy to an individual’s belief in his or her ability to organize and execute the behaviors required to manage specific tasks. When operationalized in the emotional domain, ERSE specifically pertains the degree of belief in one’s ability to effectively manage (identify, understand, regulate) both positive and negative emotional experiences. It refers to the confidence in one’s regulation abilities (efficacy belief) that directly influences his or her motivational level, behavior choice (e.g., whether to attempt to regulate), effort, and in the face of difficulties. Individuals with high ERSE are more likely to actively use adaptive emotional regulation strategies (e.g., cognitive reappraisal, problem solving) exhibit greater psychological elasticity when encountering emotional disturbances (e.g., negative emotions induced by loneliness). In the theory of emotional regulation, emotional self-efficacy is an application of the self-efficacy theory, developed on the basis of relevant theories on emotional regulation. It refers to a certain degree of self-confidence shown by individuals when dealing with their emotional states, which can relieve emotional tension, effectively relieve negative emotions (Wang and Zhao, 2015), and promote physical and mental health (Licheng et al., 2015). In addition, emotional regulation self-efficacy, a part of self-efficacy, has the quality of emotional regulation and is directly or indirectly related to individual negative emotions such as loneliness, anxiety and loss (Dou et al., 2016). Some studies also believe that emotional regulation self-efficacy is the internal mechanism of physical exercise to improve negative emotions and other negative emotions (Xu and Du, 2021; Li et al., 2013). Previous studies have proved that emotional regulation self-efficacy has a significant negative relationship with loneliness. When loneliness is used as a dependent variable, emotional regulation self-efficacy has a partial mediating effect through regression analysis through mediating effect (Cheng et al., 2020). The process of sports exercise often involves goal-setting, challenge overcoming, stress endurance, and emotional release, which are perceived by individuals as mastery experiences, one the most powerful sources for the development and enhancement of self-efficacy. Therefore, sports exercise may effectively enhance ERSE. Enhanced ERSE, in turn, endows with greater confidence and capability to manage and alleviate the negative emotions brought by loneliness and may also prompt them to seek social connections more proactively, thus, mediating the negative impact sports exercise on loneliness. This reflects the social-cognitive pathway of “behavior (exercise) → cognitive belief (ERSE) → affective state (loneliness).”Therefore, this paper proposes hypothesis H2: Physical exercise has a negative impact on loneliness through the mediating role of emotional regulation of self-efficacy.

Mental resilience is an inner positive force that helps individuals pursue their goals. Psychological elasticity does not imply that individuals are immune to stress, but rather refers to the dynamic process by which an individual maintains relatively stable physical and functioning or achieves good adaptation and recovery in the face of significant adversity, stress, or trauma. Its core lies in the protective resources that individuals possess, including both internal traits (such as optimism, self-efficacy, and cognitive flexibility) and external resources (such as social support). It emphasizes the process-oriented and malleable nature of. It involves how individuals mobilize both internal and external resources to cope effectively and adapt. High-resilience individuals typically possess superior stress appraisal abilities, a rich repertoire of coping strategies, and a sense of efficacy in recovering from setbacks. His strength is manifested in overcoming difficulties encountered in all aspects with firm faith, and enhancing self-cognition through individual growth (Sisto et al., 2019). According to the mental resilience process model, physical exercise can be regarded as a stressor, and physical exercise can make the original individual reach the physical-psychic-spiritual balance state, which can effectively regulate the individual’s behavior and emotion (Richardson, 2002; Rutter, 1985). In addition, physical exercise can improve people’s lifestyle, relieve individual negative emotions, and have a positive impact on their mental health (Zaman et al., 2019). The results of a study show that physical exercise affects the mechanism of mental resilience, and the reason is that physical exercise indirectly improves the level of mental resilience of individuals by affecting internal protection mechanism and external protection (Zhang and Qiu, 2019). Harris et al. believe that important psychological resources such as an individual’s sense of value can reduce the impact of external risk factors on individual mental health, while participating in physical exercise can promote the increase of important psychological resources and improve the level of mental resilience of individuals (Harries et al., 2019). Physical exercise can significantly improve the psychological elasticity of individuals (Zhang and Qiu, 2019). Therefore, this paper studies whether high and low levels of mental resilience play a moderating role in negative emotional experience. Psychological elasticity is conceptualized as a key individual difference variable that moderates the relationship between stressors and outcomes in this framework. Exercise itself be viewed as a positive psychophysical “resource input.” High resilience individuals, with their inherent positive qualities and resource mobilization capabilities, can more effectively identify, utilize, and the physiological, psychological, and social benefits brought by exercise, thus amplifying the buffering effect of exercise on loneliness. In contrast, low resilience individuals might struggle to fully integrate or these benefits, resulting in a blunted positive effect of exercise. This explains why the same exercise investment can produce differentiated loneliness-alleviation effects across individuals with varying resilience levels. Therefore, this paper proposes hypothesis H3: psychological elasticity positively regulates the relationship between physical exercise and loneliness. The effects of physical exercise on loneliness are not homogeneous. The individual’s psychological elasticity, which maintains or recovers good adaptation in the face of adversity, plays positive regulatory role (H3). Individuals with high psychological elasticity usually have stronger coping resources for stress.

The present study aims to deepen the understanding of the phenomenon that “exercise can reduce loneliness in college students” by empirically testing H1, H2 and H3. It is not only the core mechanism (the mediating role of emotional regulation self-efficacy) that is revealed, but also the important individual conditions (moderating role of psychological elasticity) under which it works. The findings of this study will provide new evidence about the complicated relationship between “exercise-psychological” in the field exercise psychology, and will also offer solid theoretical basis and practical guidance for designing more targeted exercise-based intervention programs for promoting the mental health and reducing loneliness among college students.

## 2 Research objects and methods

### 2.1 Research objects

The survey objects of this study were mainly selected from Shandong Huayu Institute of Technology, Yancheng Preschool Teachers College, Yulin Normal University, Zhaoqing University and Wuhan Software Vocational Engineering College, with a total of 850 undergraduates majoring in humanities and social sciences, science and technology, and art and sports. This paper strictly abides by the principle of voluntariness, and the questionnaire has been distributed online. There were 850 questionnaires in this survey. According to the screening principle, some invalid questionnaires were deleted and valid questionnaires were statistically recovered. A total of 803 valid questionnaires were obtained, with a recovery rate of 94%.

#### 2.1.1 Physical exercise scale

In this study, the Physical Activity Scale (PARS-3) revised by Liang (1994) from Wuhan Institute of Physical Education will be adopted. This scale is mainly used to measure the amount of physical activity of college students. Specifically, this scale is based on the intensity of each college student’s participation in sports activities, the duration of each exercise and the frequency of participation in sports, with the help of scoring methods to determine the amount of physical exercise, and calculate the level of physical exercise according to a specific method. It is calculated as follows: the score of physical exercise = intensity × (time-1) × frequency. Among them, time, intensity and frequency correspond to five levels respectively, and each level corresponds to a score of 1–5 points. The amount of physical activity ranged from 0 to 100 points. In addition, the amount of physical activity is divided according to the score interval: when the amount of physical activity is higher, the score is ≤19 points; when the amount of physical activity is at the medium level, the score is 20–42 points; when the amount of physical activity is low, the score is ≥43 points. The Cronbach ‘salpha value of this study reached 0.659. At present, this scale is mainly applied to the physical activity measurement of college students and other groups.

#### 2.1.2 UCLA loneliness scale

UCLA loneliness scale third edition (UCLALonelinessScaleVersion3) was established in 1996 by Russell and others develop into the scale (Russel, 1996). This scale is mainly used to investigate the subjective loneliness caused by the gap between the expectation of social interaction and the actual level of social interaction. There are a total of 20 items in this scale, including 11 items for forward scoring and 9 items for reverse scoring. The scoring method adopts five-point scoring method, and the total score of the scale is positively correlated with the degree of loneliness felt by an individual, that is, the higher the total score, the higher the degree of loneliness felt by an individual (Rutter, 1985). In this study, the internal consistency reliability coefficient of the scale was 0.971.

#### 2.1.3 Emotional regulation self-efficacy scale

This scale is the Emotional Regulation Self-efficacy Scale (RES), compiled by Caprara et al., and later revised by Shu et al. (2009). The scale covers three dimensions: positive emotion expression efficacy (POS), frustration/distress emotion regulation efficacy (DES) and anger/anger emotion regulation efficacy (ANG). The scale contains a total of 12 items, which are scored using the Likert five-point scale, with the total score ranging from 12 to 60 points. Among them, the higher the total score, the stronger the individual’s ability in emotional regulation self-efficacy. In this study, the Cronbach’sα coefficient of this scale was 0.883.

#### 2.1.4 Psychological elasticity scale

This paper uses the “The Connor-Davidson Resilience Scale” (CD-RISC) compiled by Connor and Davidson (2010). The scale has been revised by scholars such as Yu Xiaonan and Zhang Jianxin, including three dimensions of resilience, strength and optimism. Among them, the optimism dimension has 4 items, namely items 2, 3, 4 and 6; The strength dimension includes 8 items, namely items 1, 5, 7, 8, 9, 10, 24 and 25. The toughness dimension has 13 items, which are items 11-23. There are 25 items in the scale. This scale adopts Likert 5-point scoring method, the higher the score, the stronger the psychological elasticity of the individual. In this study, the Cronbach’s alpha value of this scale reached 0.975, which is currently mainly used to measure mental resilience of college students and other groups.

### 2.2 Common method deviation test

In this study, Harman single factor test was used to test the common method bias. The results show that there are 7 factors with characteristic roots greater than 1, and the first factor explains the cumulative variation of 39.156%. As long as it is below the critical value of 40%, there is no serious common method bias problem.

## 3 Research results

### 3.1 Correlation coefficients of each variable

The average scores of each variable were correlated, and the results showed that physical exercise had a significant negative correlation with loneliness. Physical exercise was positively correlated with self-efficacy of emotion regulation. There was a significant negative correlation between emotional self-efficacy and loneliness. Physical exercise is positively correlated with mental resilience. There was a significant negative correlation between psychological elasticity and loneliness (Table 1).

**Table 1:** Correlation coefficient matrix of each variable.

Variable name	Exercise intensity	Exercise time	Exercise frequency	Loneliness	Vigorous	Dejected	Get angry	Optimism	Strength	Tenacity
Exercise intensity	1									
Exercise time	0.434**	1								
Exercise frequency	0.400**	0.368**	1							
Loneliness	−0.410**	−0.374**	−0.344**	1						
Vigorous	0.233**	0.197**	0.185**	−0.373**	1					
Dejected	0.265**	0.225**	0.174**	−0.339**	0.293**	1				
Get angry	0.224**	0.202**	0.152**	−0.390**	0.329**	0.331**	1			
Optimism	0.243**	0.179**	0.209**	−0.436**	0.400**	0.266**	0.325**	1		
Strength	0.308**	0.203**	0.254**	−0.352**	0.432**	0.281**	0.292**	0.561**	1	
Tenacity	0.385**	0.314**	0.331**	−0.483**	0.520**	0.441**	0.369**	0.523**	0.624**	1

* represents *p* < 0.05, ** represents *p* < 0.01, *** represents *p* < 0.001.

### 3.2 Direct effect analysis

**Figure 1:**
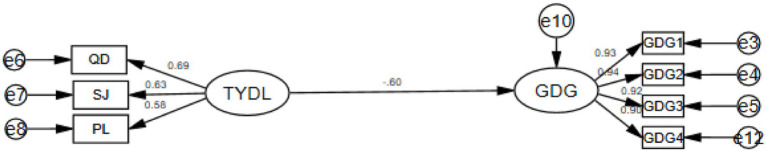
Model of direct effect of physical exercise on loneliness. TYDL, physical exercise; XXTR, learning engagement; QD, intensity; SJ, time; PL, frequency; GDG1-GDG4, The four variables packaged in the loneliness project; e, error term.

The direct impact model of physical exercise on loneliness was built with the help of AMOS28.0 software. After setting the correlation and residual among variables, the corresponding results were obtained, as shown in the Figure 1.

After the direct effect model is constructed, the fit degree and fit degree of the model are tested. For details, please refer to the following table.

As can be seen from Table 2, the actual value of χ2/df is 3.277 and the value of RMSEA is 0.053. According to relevant standards, the requirement is met when the value of RMSEA is lower than 0.1. In addition, RFI, NFI, TLI, IFI, and CFI values are all greater than 0.9. This shows that the model has a good fitting degree.

**Table 2:** Structural equation model fitting index.

Adaptation index	^χ2^/df	RMSEA	NFI	IFI	TLI	CFI	RFI
Actual value	3.277	0.053	0.993	0.984	0.993	0.989	0.993

As can be seen from the Table 3, the standardization coefficient is −0.639 (*p* < 0.001), indicating that physical exercise has a significant negative impact on loneliness.

**Table 3:** Direct effect path coefficient estimation table.

Path	Non-standardized coefficients	Standardization coefficient	S. E	*t*	*p*
Physical exercise → Loneliness	0.587	0.650	0.046	12.646	0.000

### 3.3 Intermediation effect test

**Figure 2:**
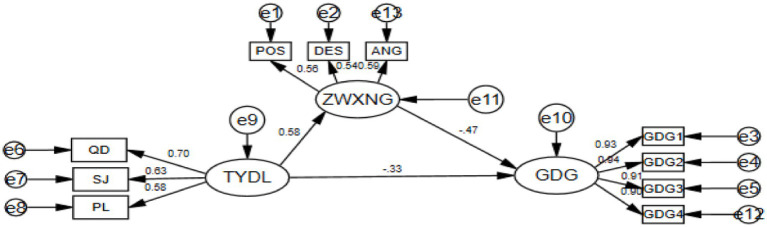
The mediating mechanism model of physical exercise on loneliness. TYDL, physical exercise; ZWXNG, Emotional self-regulation efficacy; ZWKZ, self-control; QD, intensity; SJ, time; PL, frequency; POS, vigorous; DES, dejected; ANG, Get angry; GDG1-GDG4, The four variables packaged in the loneliness project; e, error term.

In this study, structural equation model is used to analyze the relationship between variables. Specifically, first of all, we refer to Wen and Ye (2014) for the analysis method of mediating effect model test, and test the mediating model with the help of Amos28.0 software. In the process of inspection, the percentile Bootstrap method of bias correction was used to repeat 5,000 samples, and a 95% confidence interval was calculated accordingly. Finally, the intermediary model was successfully established, as shown in Figure 2.

As can be seen from Table 4, the value of χ^2^/df is 2.611, the value of RMSEA is 0.045, which is less than 0.080, and the values of NFI, TLI, IFI and CFI are all greater than 0.9, indicating that the model is well fitted.

**Table 4:** Fitting index of structural equation model.

Adaptation index	χ^2^/df	RMSEA	NFI	IFI	TLI	CFI	RFI
Actual value	2.611	0.045	0.982	0.975	0.989	0.985	0.989

According to Table 5, the standardized coefficient of the path “physical exercise → emotional regulation self-efficacy” is 0.580 (*p* < 0.001), indicating that physical exercise has a significant positive impact on emotional regulation self-efficacy. The standardized coefficient of the path “physical exercise → loneliness” was −0.329 (*p* < 0.001), indicating that physical exercise had a significant negative effect on loneliness. The standardized coefficient of the path “emotional regulation self-efficacy → loneliness” was −0.474 (*p* < 0.001), indicating that emotional regulation self-efficacy had a significant negative impact on loneliness.

**Table 5:** Path coefficient estimation table.

Path	Non-standardized coefficients	Standardization coefficient	S. E	*t*	*p*
Physical Exercise → Emotional regulation Self-efficacy	0.340	0.580	0.040	8.442	0.000
Physical exercise → Loneliness	−0.345	−0.329	0.060	−5.719	0.000
Emotional regulation Self-efficacy → loneliness	−0.848	−0.474	0.120	−7.082	0.000

*** stands for *p* < 0.001.

As can be seen from Table 6, the direct effect value of physical exercise on loneliness is −0.361, and bias-corrected 95% CI confidence interval is [−0.422, −0.300], Percentile 95%CI confidence interval [−0.422, −0.300], 0 is excluded. The direct effect is significant. The indirect effect of physical exercise on loneliness through emotional regulation of self-efficacy was −0.131. The results of the mediation effect test based on the Bootstrap method showed that the bias-corrected 95%CI interval was [−0.166, −0.095], and the percentile 95% CI confidence interval [−0.169, −0.097], excluding 0. It shows that the mediating effect is significant. The total effect value of physical exercise on loneliness was −0.492, the bias-corrected 95% CI confidence interval was [−0.553, −0.431], and the percentile 95% CI confidence interval [−0.553, −0.431], excluding 0, indicated that the total effect was significant. Therefore, emotional self-efficacy plays a partial mediating role between physical exercise and loneliness.

**Table 6:** Tests the mediating effect of emotional regulation on self-efficacy.

Path	Point estimate	Product of coefficients		Boostrap 5,000 times			
				Bias-corrected 95%CI		Percentile 95%CI	
		Standard error	Z value	Floor	Upper limit	Floor	Upper limit
The total effect of physical exercise on loneliness	−0.492	0.031	−15.771	−0.553	−0.431	−0.553	−0.431
Physical exercise → Emotional regulation self-efficacy → loneliness into indirect effects	−0.131	0.018	−7.306	−0.166	−0.095	−0.169	−0.097
The direct effect of physical exercise on loneliness	−0.361	0.031	−11.624	−0.422	−0.300	−0.422	−0.300

Based on the above results, this study proposed H1 and H2 hypotheses on the direct and mediating effects of physical exercise on loneliness, and the H1 and H2 hypotheses were verified by testing, as shown in the following Table 7.

**Table 7:** Results of hypothesis verification of direct action and mediation mechanism.

Hypothetical number	Hypothetical content	Test result
H1	Physical exercise has a negative effect on loneliness	support
H2	Physical exercise has a positive effect on loneliness through the mediating effect of emotion regulating self-efficacy	support

### 3.4 Adjustment effect test

Based on the existing literature, this study introduced moderating variables to explore the impact of physical exercise on loneliness. At the same time, four variables, such as gender, grade, major and place of origin, were selected as control variables. In the research process, SPSS27.0 software was used to carry out statistical work on relevant data, and PR0CESS software was used to carry out inspection, as shown in the following table for details.

According to the results of Table 8, the *β* value and T-value coefficient of grade were significant (*p* < 0.05), but the β value and T-value coefficient of gender, grade, major and student origin were not significant (*p* > 0.05). By examining the main effect, physical exercise had a significant negative impact on loneliness (*β* = −0.341, *p* < 0.01), and mental resilience had a significant negative impact on loneliness (*β* = −0.330, *p* < 0.01). By testing the regulating effect of mental resilience, it can be seen that the influence of “physical exercise × mental resilience” on loneliness is −0.156 (*p* < 0.01), indicating that mental resilience has a positive regulating effect between physical exercise and loneliness, that is, physical exercise has a negative influence on loneliness, and the interaction term is also a negative influence, which is a positive adjustment (Table 9).

**Table 8:** Test of adjustment effect of mental resilience.

Regression equation (*N* = 803)		Fitting index			Coefficient significance	
Result variable	Predictor	*R*	*R^2^*	*F(df)*	*β*	*T*
Loneliness		0.607	0.369	66.367		
	Sex				−0.033	−1.154
	Grade				0.048	1.669
	Profession				0.047	1.637
	Source of students				0.024	0.825
	Physical exercise				−0.341**	−10.609
	Mental resilience				−0.330**	−10.374
	Physical exercise x mental resilience				−0.156**	−5.450

“×” represents the cross-multiplication term, * represents *p* < 0.05, *** represents *p* < 0.00.

**Table 9:** Results of hypothesis verification of the regulatory mechanism.

Suppose the number	Suppose the content	Result
H3	Psychological elasticity positively regulates the relationship between physical exercise and loneliness.	support

## 4 Discussion

### 4.1 The direct effect of physical exercise on loneliness

It is found that physical exercise has a significant negative predictive effect on loneliness of college students, which is consistent with the research results of Quan (2012) and Bai (2023). It shows that physical exercise affects the degree of loneliness of college students.

A large number of literatures have confirmed that physical exercise plays a positive role in improving individual physical and mental health (Kim, 2015). Loneliness, as a negative emotional state, has a profound and non-negligible impact on individual mental health. For this reason, in-depth research on how physical exercise can improve mental health by reducing loneliness and its various dimensions becomes a very meaningful and necessary work. Moreover, physical exercise can also help individuals improve their social communication skills, so that they can show stronger self-confidence in other different social scenarios, so as to further reduce loneliness and optimize the quality of relationship connection (Barbieri, 2021). Self-esteem and self-efficacy are undoubtedly essential elements of a mental health system. Physical exercise can have a positive impact on an individual’s body image and self-evaluation. When an individual successfully achieves the established sports goals, his inner sense of achievement and self-confidence will be greatly enhanced, and then the sense of loneliness will be effectively reduced, especially in the dimension of alienation (Fox, 1999). From the physiological level of analysis, the many benefits brought by physical exercise, the positive impact on mental health can not be underestimated. Regular and moderate physical activity has a significant effect on improving individual sleep quality, which can help people to relieve the daily stress and anxiety, reduce the probability of depression symptoms, and then reduce the overall loneliness and the impact of various dimensions. After all, good physical condition is always a solid foundation for maintaining mental health (Yali et al., 2020).

In summary, it can be seen that the results of this study strongly support the theoretical hypothesis that physical exercise can significantly reduce loneliness and its various dimensions, and effectively promote individual mental health through this specific mechanism of action.

### 4.2 The mediating role of emotional regulation of self-efficacy

For example, Wang Kun et al. conducted a survey on self-efficacy of exercise and emotion regulation. The results of the study clearly showed that there was a significant positive relationship between physical exercise and emotional regulation self-efficacy. Based on this, they believe that active participation in sports can help college students improve their emotional regulation self-efficacy and relieve anxiety, so that they can better manage their emotions (Hausenblas and Janelle, 2001). The mediating effect of emotion regulation self-efficacy is not limited to the context of sports exercise, and it can last by influencing the choice of emotion strategies (such as cognitive reappraisal takes precedence over expressive suppression) to reduce loneliness. Train physical education teachers, club coaches, and counselors to embed emotional regulation teaching into their guidance of sports. For example, guide reflection after exercise on “How cope with training setbacks/pressure”; "Transfer the persistence in sports to emotion management.” Ouyangy et al.’s study shows that the higher the level of college students’ participation in physical exercise, the corresponding sense of sports self-efficacy will be enhanced (Sherwood et al., 2004). Physical exercise can directly enhance individuals’ belief in their ability to regulate emotions through a “goals achievement-role modeling-feedback reinforcement” cycle, forming aexercise behavior → self-efficacy →loneliness alleviation” transmission path. The study of Downs et al. showed that with the gradual increase of exercise duration, emotional regulation self-efficacy would also increase (Wang et al., 2022). In addition, some studies focusing on adolescent females have similar findings, that is, adolescent girls who participate in physical activities generally have higher self-efficacy than girls who do not participate in any physical activities (Ouyang et al., 2019). A number of other studies have pointed out that moderate to vigorous aerobic exercise will have a significant impact on emotional regulation self-efficacy (Hamilton et al., 2017). According to McAuley et al., participation in aerobic exercise programs may optimize mental and physical conditions, thereby promoting the improvement of self-efficacy (Flanagan and Perry, 2018). Results by Lubans et al. have shown that adolescents who participate in resistance training have superior performance in emotional regulation self-efficacy compared to the control group (Garcia-Silva et al., 2018). Although there is a lot of evidence showing a significant correlation between sports and emotional regulation self-efficacy, in fact, there may be a mutually reinforcing relationship between the two (Mcauley et al., 1991). That is, although physical exercise may have an improved effect on emotional regulation self-efficacy, emotional regulation self-efficacy may also prompt subjects to engage more in physical activity. It has been proved that a healthy mental state can enhance people’s intention to participate in sports (Lubans et al., 2010). People with lower self-efficacy of emotion regulation are more likely to have more serious negative emotions (Sallis et al., 2000). Studies have found that emotional regulation self-efficacy is an important factor affecting mental health, which can directly predict loneliness experience, and the decrease of emotional regulation self-efficacy can directly deepen loneliness (Li et al., 2013). Explain that physical exercise, by increasing ERSE, prompts individuals to adopt more “problem-focused strategies” rather than “emotion-focused strategies,” fundamentally the occurrence of loneliness. As a dependent variable, emotional self-efficacy plays a partial mediating role in psychological abuse and loneliness (Cheng et al., 2020).

In summary, the results of this study confirm the research hypothesis that physical exercise can not only directly affect the loneliness experience of college students, but also affect their loneliness experience by improving their emotional regulation self-efficacy.

### 4.3 The regulating effect of mental resilience

First of all, psychological elasticity is one of the manifestations of psychological capital, which can play a role of buffer in any stress state (Juzhe, 2014). College students with low mental resilience can bear it. The results show that psychological elasticity plays a significant role in the adjustment of physical exercise on the loneliness experience of college students. Among them, in the path of the model, it is shown that compared with college students with high mental resilience, the reduction of loneliness of college students with low mental resilience decreases significantly with the increase of physical exercise. Therefore, physical exercise has a significant easing effect on the loneliness of college students with low mental resilience. Psychological elasticity, as a dynamic capacity (rather than a stable trait), amplifies the mitigating effect of exercise on loneliness under the continuous stimulation physical exercise through a spiral-upward process of “stress exposure → coping strategy optimization → resource accumulation.” Integrate a simple resilience screener into admissions or annual psychological assessments. For low resilience groups, provide: “Exercise “enhanced support: Small- coaching-style exercise classes, with psychologists on hand to provide emotional support and social skills training. This research result also confirms the theoretical model of psychological capital (Li et al., 2014), which holds that psychological capital is one of the important factors affecting an individual’s psychological state. When an individual has a higher psychological capital, the chances of mental health problems such as anxiety, loneliness and depression are smaller. On the contrary, when an individual has a lower psychological capital, the chances of mental health problems such as anxiety, loneliness and depression are smaller. The greater the chance of developing mental health problems (Chong et al., 2019). In addition, from the perspective of indirect path analysis, when negative emotions continue to rise, the rise rate of negative emotions of college students with low mental resilience level is significantly higher than that of college students with high mental resilience level. Finally, this phenomenon once again strongly proves that psychological elasticity plays an important role in regulating the relationship between physical exercise and loneliness of college students, and psychological elasticity can effectively enhance the influence of physical exercise on loneliness. It can be seen that improving the mental resilience of college students in various ways can enable them to maintain a relatively good mental state even when facing high difficulties, thus effectively reducing the incidence of loneliness. It is worth noting that recent relevant research results show that sports play a positive role in improving the psychological elasticity of individuals (Eory et al., 2021). Based on this, sports can not only directly reduce the loneliness of individuals, but also indirectly play an anti-anxiety effect by virtue of the mediating role of stress perception (Lago et al., 2019). More importantly, sports can also buffer the impact of stress stimuli or stressful events on mental health by enhancing individual psychological elasticity (Zi et al., 2019), thus reducing individual loneliness to a greater extent. Therefore, college students with higher level of mental resilience can alleviate individual loneliness and enhance their mental health during physical exercise.

## 5 Conclusion and suggestion

### 5.1 Conclusion

This paper takes the loneliness of college students as the research background, and based on the self-efficacy of emotional regulation [54], introduces the self-efficacy of emotional regulation to build the mediating mechanism model of physical exercise on loneliness. At the same time, taking psychological elasticity as the regulating variable, this paper analyzes the regulating mechanism of physical exercise on loneliness, and through correlation analysis and structural equation model and other statistical methods, the following conclusions are obtained:

(1) Physical exercise of college students has a significant negative impact on loneliness. Physical exercise has a positive effect on loneliness through the mediating effect of emotion regulating self-efficacy psychological elasticity positively moderates the relationship between physical exercise and loneliness.
(2) Through the theoretical analysis of how physical exercise can improve the loneliness of college students, a new path for physical exercise to alleviate the loneliness of college students is established by introducing emotional regulation self-efficacy as the mediating variable and psychological elasticity as the regulating variable.
(3) Through research, it is found that college students’ active participation in physical exercise can improve the self-efficacy of emotional regulation, adjust physical exercise through psychological elasticity, and alleviate the loneliness of college students. It shows that the mediating effect of self-efficacy and the regulating effect of mental resilience are valid.
(4) The results show that the direct predictive effect of physical exercise on college students’ loneliness is significant (*β* = −0.600, *p* < 0.001). The results of mediation effect analysis show that the standardized coefficient of the path “physical exercise → emotional regulation self-efficacy” is 0.580 (*p* < 0.001), and the standardized coefficient of the path “emotional regulation self-efficacy→loneliness” is −0.740 (*p* < 0.001); psychological elasticity plays a partial mediating role between physical exercise and loneliness in college students (*β* = −0.150, *p* < 0.01).

### 5.2 Suggestion

The research results of this article show that physical exercise has a significant negative impact on its sense of loneliness, and that physical exercise reduces loneliness through the partial mediation of regulation self-efficacy. Resilience plays a positive regulatory role in the relationship between physical exercise and loneliness. According to this result, the following suggestions are put forward in article:

(1) Colleges and universities should fully realize the positive effect of physical exercise on loneliness, strengthen the intervention of physical exercise, organize more sports-related activities competitions, ensure the opening of sports venues, organize more sports activities and sports competitions, and actively carry out college associations as a second classroom, so that college students can actively participate physical exercise, and improve self-control ability.
(2) The samples may not fully represent the student population of different types of institutions. Differences in resource endowments, campus culture, sports facilities, and student compositions (e.g., urban–rural background, economic background) across institutions may influence exercise patterns, levels of resilience, and loneliness experiences. To enhance the generalizability the findings, future studies will employ stratified sampling or multi-stage sampling methods to include a more diverse range of higher education institutions and a broader geographic distribution of colleges and universities.active examination and reporting of potential influences of background variables such as types of institutions and geographical regions on the central research models (H1, H2, and H3) in the analysis be conducted to more accurately delineate the boundary conditions of research findings.
(3) Although this study controlled for gender, grade, and major, it is important to acknowledge that other potential confounding factors were not systematically measured or controlled. In the, key confounders will be actively measured and controlled, and the temporal relationship and unique contributions of the variables will be more rigorously examined using longitudinal designs or statistical controls (e.g., covariate analysis).

## Data Availability

The original contributions presented in the study are included in the article/Supplementary material, further inquiries can be directed to the corresponding author.
